# Comparative effectiveness of different corticosteroid regimens in severe alcohol-associated hepatitis

**DOI:** 10.1097/HC9.0000000000000573

**Published:** 2024-10-24

**Authors:** Alvi Husni Islam, Luis Antonio Díaz, Francisco Idalsoaga, Leonardo Guizzetti, Rokhsana Mortuza, Winston Dunn, Ashwani K. Singal, Douglas Simonetto, Carolina Ramirez-Cadiz, Wei Zhang, Steve Qian, Joaquín Cabezas, Shiv K. Sarin, Rakhi Maiwall, Prasun K. Jalal, Fatima Higuera-De La Tijera, Lubomir Skladany, Natalia Bystrianska, Diego Rincon, Kristina R. Chacko, Meritxell Ventura Cots, Guadalupe Garcia-Tsao, Juan G. Abraldes, Patrick S. Kamath, Marco Arrese, Vijay Shah, Ramon Bataller, Juan Pablo Arab

**Affiliations:** 1 Department of Medicine, Division of Gastroenterology, Schulich School of Medicine, Western University & London Health Sciences Centre, London, Ontario, Canada; 2 Departamento de Gastroenterología, Escuela de Medicina, Pontificia Universidad Católica de Chile, Santiago, Chile; 3 MASLD Research Center, Department of Medicine, Division of Gastroenterology and Hepatology, University of California San Diego, San Diego, California, USA; 4 Maple Statistical Consulting, Ottawa, Ontario, Canada; 5 Department of Medicine, Division of Gastroenterology, University of Kansas Medical Center, Kansas City, Kansas, USA; 6 Department of Medicine, Division of Gastroenterology, Hepatology and Nutrition, University of Louisville School of Medicine, Louisville, Kentucky, USA; 7 Department of Medicine, Division of Gastroenterology and Hepatology, Mayo Clinic, Rochester, Minnesota, USA; 8 Department of Anesthesia, Virginia Commonwealth University School of Medicine, Richmond, Virginia, USA; 9 Gastroenterology Unit, Department of Medicine, Massachusetts General Hospital, Harvard Medical School, Boston, Massachusetts, USA; 10 Department of Medicine, Division of Gastroenterology and Hepatology, University of Florida, Gainesville, Florida, USA; 11 Gastroenterology and Hepatology Department, University Hospital Marqués de Valdecilla, Research Institute Valdecilla (IDIVAL), Santander, Spain; 12 Department of Hepatology, Institute of Liver and Biliary Sciences, New Delhi, India; 13 Department of Gastroenterology and Hepatology, Baylor College of Medicine, Houston, Texas, USA; 14 Servicio de Gastroenterología, Hospital General de Mexico, Universidad Nacional Autónoma de Mexico, Ciudad de Mexico, Mexico; 15 Department of Internal Medicine II, Division of Hepatology, Gastroenterology and Liver Transplantation, Slovak Medical University, F.D. Roosevelt University Hospital, Banska Bystrica, Slovak Republic; 16 Liver Unit, Department of Digestive Diseases, Hospital General Universitario Gregorio Marañón, CIBEREHD Centro de Investigación Biomédica en Red de Enfermedades Hepáticas y Digestivas, Madrid, Spain; 17 Division of Gastroenterology and Hepatology, Montefiore Medical Center, Bronx, New York, USA; 18 Liver Unit, Hospital Vall d’Hebron, Universitat Autonoma Barcelona, CIBEREHD, Barcelona, Spain; 19 Section of Digestive Diseases, Yale University School of Medicine/VA-CT Healthcare System, New Haven/West Haven, Connecticut, USA; 20 Department of Medicine, Division of Gastroenterology, Liver Unit, University of Alberta, Edmonton, Alberta, Canada; 21 Liver Unit, Hospital Clinic, Barcelona, Spain; 22 Department of Medicine, Institut d’Investigacions Biomèdiques August Pi i Sunyer (IDIBAPS), Barcelona, Spain; 23 Department of Internal Medicine, Division of Gastroenterology, Hepatology, and Nutrition, Virginia Commonwealth University School of Medicine, Richmond, Virginia, USA

**Keywords:** alcohol, alcohol-associated cirrhosis, alcohol-associated hepatitis, alcohol-associated liver disease, ethanol, steroids

## Abstract

**Figure:**
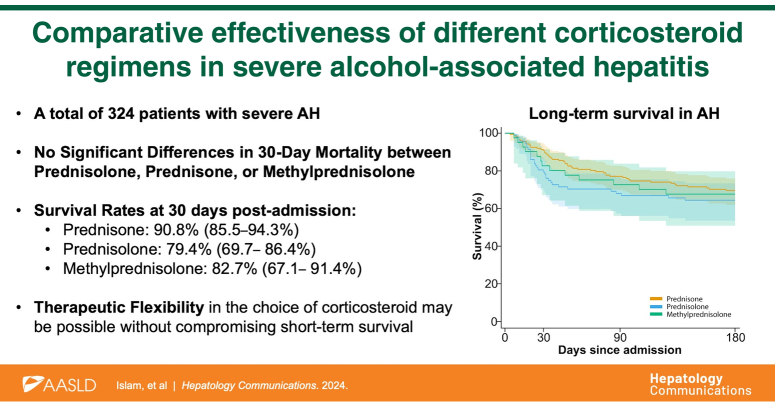

## INTRODUCTION

Severe alcohol-associated hepatitis (AH) is a life-threatening condition characterized by significant liver inflammation and dysfunction, leading to high mortality rates.^1^ Despite advancements in medical management, AH remains a clinical challenge, with limited therapeutic options that effectively improve patient outcomes. Corticosteroids, particularly prednisolone, have long been the cornerstone of treatment for severe AH due to their potent anti-inflammatory properties and reduction in short-term mortality.^2^ Prednisolone has been preferred over other corticosteroids due to its favorable safety profile and the theoretical benefit of not requiring hepatic metabolism to become active.^3^ Subsequent efforts have been made to clarify the therapeutic window and benefit from corticosteroid use, identifying that those with a MELD score over 20, but especially those between 25 and 39, have lower 30-day mortality.^4^

Comparative studies between corticosteroid types, in the context of severe AH, are limited. The major differences between these corticosteroids lie in their pharmacokinetics, mode of delivery, metabolism, and potential side effects. Understanding these differences is crucial for optimizing treatment strategies, especially in settings where specific corticosteroids may not be readily available.^5^ As such, this study aimed to compare the most used corticosteroid types, prednisone, prednisolone, and methylprednisolone, to treat AH.

## METHODS

This retrospective international multicenter cohort study included adults with a clinical and/or histological diagnosis of severe AH. Data were collected from multiple centers across different countries, ensuring a diverse and representative sample. Data were derived from 324 patients, focusing on the 3 most used steroids: prednisone, prednisolone, and methylprednisolone. The primary outcome measured was time to death. Secondary outcomes included 30-, 90-, and 180-day mortality.

Time to death was evaluated using Kaplan-Meier (KM) curves by steroid type, as well as Cox proportional hazards regression models. Survival rates were estimated from KM curves at 30, 90, and 180 days after admission. The pseudo-values approach was used to examine differences in the fixed-time 30-day survival estimates using the complementary-log-log link.^6^ These survival estimates and Cox regression models were adjusted for using a predefined set of established predictors of mortality in AH from previous literature, including age, sex, history of cirrhosis, acute kidney injury at admission, and MELD score at admission. Statistical significance was based on a 2-sided *p* < 0.05.

## RESULTS

The study included 324 patients with severe AH. The baseline demographic and clinical characteristics of the sample can be found in Supplemental Table S1, http://links.lww.com/HC9/B69. The median age was 47.3 ± 11.2 years, and 69.2% of the patients were male. The median MELD score at admission was 24 [21–28]. Among the participants, 43% received corticosteroid treatment, distributed as follows: 55.6% (186 patients) used prednisone, 27.5% (95 patients) used prednisolone, and 14.1% (43 patients) used methylprednisolone. Significant differences were observed in age (*p* < 0.01), admission MELD score (*p* = 0.01), admission bilirubin (*p* = 0.01), admission creatinine (*p* = 0.02), and cirrhotic status (*p* < 0.01), across the 3 steroid groups. Based on Lille score on day 4 or day 7, there were no significant differences among these 3 corticosteroids (prednisone 55.4% vs. prednisolone 50.5% vs. methylprednisolone 44.2%, *p* = 0.38) (Supplemental Table S1, http://links.lww.com/HC9/B69).

In the multivariable regression analysis for time to death, there were no significant differences between the corticosteroid types. Compared to prednisolone, prednisone (HR: 0.73, 95% CI: 0.49–1.08; *p* = 0.12) and methylprednisolone (HR: 0.88, 95% CI: 0.53–1.45; *p* = 0.61) were not statistically significantly associated with mortality. However, age (HR: 1.27, 95% CI: 1.09–1.48; *p* < 0.01) and MELD score (HR: 1.06, 95% CI: 1.03–1.09; *p* < 0.01) were statistically significantly associated with mortality.

**FIGURE 1:**
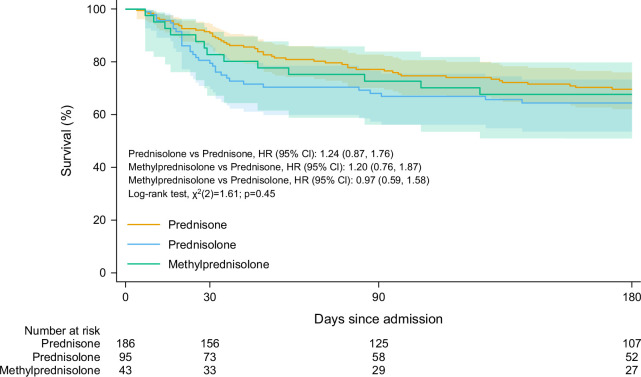
Kaplan-Meier curve of survival in patients with severe alcohol-associated hepatitis by steroid type (prednisone, prednisolone, and methylprednisolone).

The KM survival curve and estimates for each steroid type are demonstrated in Figure 1 and Supplemental Table S2, http://links.lww.com/HC9/B69. There was no evidence of overall differences in survival by steroid type (log-rank *p* value = 0.45). In terms of survival at a fixed time, KM survival estimates at 30 days after admission showed no significant differences between steroid types (*p* = 0.12). The adjusted KM survival probability estimates were 79.4% (95% CI: 69.7%–86.4%) for prednisolone, 90.8% (95% CI: 85.5%–94.3%) for prednisone, and 82.7% (95% CI: 67.1%–91.4%) for methylprednisolone. The relative risk of 30-day survival for prednisone compared to prednisolone was 1.51 (95% CI: 0.85–2.68; *p* = 0.16), and for methylprednisolone compared to prednisolone was 1.10 (95% CI: 0.57–2.10; *p* = 0.77). The absolute risk difference, compared to prednisolone, was 9.9 percentage points (pp) (95% CI: −0.2 pp–20.0 pp; *p* = 0.05) for prednisone and 4.2 pp (95% CI: −9.7 pp–18.1 pp; *p* = 0.55) for methylprednisolone.

## DISCUSSION

This study found no statistically significant differences between prednisolone, prednisone, and methylprednisolone in predicting time to death among patients with severe AH. These findings suggest that the choice of corticosteroids may not influence short-term clinical outcomes in this patient population, highlighting the potential for flexibility in therapeutic regimens based on availability, cost, and patient-specific factors. To our knowledge, no head-to-head trials comparing steroid types in studying survival outcomes in AH exist; therefore, this should be included in future research directions.

The observed lack of significant difference among the corticosteroids aligns with some previous research. Previously, a meta-analysis including 6 studies concluded that the effectiveness of corticosteroids in reducing short-term mortality in severe AH is consistent across different types, reinforcing the notion that the anti-inflammatory properties of corticosteroids are the critical factor rather than the specific steroid used.^3^ Our study extends these findings by directly comparing different corticosteroids and reinforcing that age and MELD score remain critical predictors of mortality and response to treatment in AH. These predictors should be carefully considered when managing patients with severe AH. In the future, validation of new models to determine the benefit of pharmacological therapies could help guide clinicians in prescribing corticosteroids and better identify who should be referred early for liver transplantation.^7^,^8^

Despite the robustness of our multicenter cohort study, some limitations must be acknowledged. The retrospective nature of the study may introduce biases related to patient selection and treatment allocation. In addition, variations in clinical practice and corticosteroid dosing protocols across different centers might have influenced the outcomes.^4^ Adverse events from corticosteroids, such as infections occurring at the end of follow-up, could also inform decision-making; however, they were not recorded in this study. Finally, alcohol abstinence and treatment of alcohol use disorder were not considered in this analysis; however, this is a relevant aspect in the survival of patients with AH.^9^,^10^ Thus, further studies in AH should include alcohol use disorder management as a relevant and complementary strategy in the management of patients with severe AH.

In conclusion, while our study did not find a significant difference in the effectiveness of the 3 most used corticosteroids for short-term outcomes in severe AH, it contributes valuable data to the ongoing discourse on optimizing treatment strategies. Future, longitudinal and randomized clinical research should continue to explore the intricate dynamics of corticosteroid therapy, aiming for personalized and more effective management of severe AH.

## Supplementary Material

## Data Availability

The data that support the findings of this study are available from the corresponding author upon reasonable request. Marco Arrese receives support from the Chilean government through the Fondo Nacional de Desarrollo Científico y Tecnológico (FONDECYT #1241450). Ashwani K. Singal consults and owns stock in Pleiogenix. He advises and is on the speaker’s bureau for the CLD Foundation. He consults for AGA. He advises Durect, Gastroendo News, and AASLD. He is on the speaker’s bureau for Medscape Gastroenterology. He received grants from NIH. Douglas Simonetto consults for Mallinckrodt, BioVie, Pharma Inc., Resolution Therapeutics, Iota, Evive, and AstraZeneca. Joaquin Cabezas advises and received grants from Gilead. He received grants from Abbvie. Lubomir Skladany consults, advises, is on the speaker’s bureau, and received grants from Astellas. He consults, advises, and is on the speaker’s bureau for Gilead. He is on the speaker’s bureau and received grants from Promed and WORWAG. He received grants from FALK. Juan G. Abraldes consults for 89bio, Boehringer Ingelheim, AstraZeneca, Agomab, Boston Pharmaceuticals, and Novo Nordisk. He received grants from Cook and Gilead. Ramon Bataller consults for GlaxoSmithKline, Novo Nordisk, Boehringer Ingelheim, and Resolution Therapeutics. He is on the speaker’s bureau for Gilead and Abbvie. The remaining authors have no conflicts to report. An informed consent waiver was obtained at each participating center, and the data were analyzed and deidentified.
