# Supplementary material for: Comparative effectiveness of different corticosteroid regimens in severe alcohol-associated hepatitis
Source: Hepatol Commun. 2024 Oct 24;8(11):e0573. doi: 10.1097/HC9.0000000000000573 (PMC11512629; doi:10.1097/HC9.0000000000000573)
Supplement: Supplementary file 1 [file hc9-8-e0573-s001.docx]

**Supplementary Table 1.** Baseline and clinical characteristics among recent corticosteroid users

|  | **Prednisone (n=186)** | **Prednisolone (n=95)** | **Methylprednisolone (n=43)** | **Overall (n=324)** | **P-Value ^a^** |
| --- | --- | --- | --- | --- | --- |
| Male (%) | 115/186 (61.8%) | 63/95 (66.3%) | 32/43 (74.4%) | 210/324 (64.8%) | 0.28 |
| Age at admission (years) | 49.8 (9.9) | 44.5 (11.0) | 49.6 (11.0) | 48.2 (10.6) | <0.01 |
| Age at admission < 40 years | 32/186 (17.2%) | 31/95 (32.6%) | 9/43 (20.9%) | 72/324 (22.2%) | 0.01 |
| Age at admission 40 to <65 years | 142/186 (76.3%) | 59/95 (62.1%) | 30/43 (69.8%) | 231/324 (71.3%) | 0.04 |
| Age at admission ≥ 65 years | 12/186 (6.5%) | 5/95 (5.3%) | 4/43 (9.3%) | 21/324 (6.5%) | 0.67 |
| Admission MELD score | 24.6 (5.2) | 26.9 (7.7) | 26.6 (7.5) | 25.5 (6.4) | 0.01 |
| Admission total bilirubin | 15.5 (9.3) | 19.2 (11.4) | 18.7 (10.0) | 17.0 (10.2) | 0.01 |
| Admission serum albumin | 2.7 (0.6) | 2.7 (0.6) | 2.6 (0.5) | 2.7 (0.6) | 0.75 |
| Admission creatinine | 1.0 (0.6) | 1.3 (1.4) | 1.3 (1.4) | 1.1 (1.1) | 0.02 |
| Infection, Yes vs No | 51/137 (37.2%) | 4/10 (40.0%) | 13/27 (48.1%) | 68/174 (39.1%) | 0.57 |
| Cirrhotic, Yes vs No | 154/183 (84.2%) | 62/94 (66.0%) | 35/43 (81.4%) | 251/320 (78.4%) | <0.01 |
| AKI at admission, Yes vs No | 43/186 (23.1%) | 28/95 (29.5%) | 11/43 (25.6%) | 82/324 (25.3%) | 0.51 |
| Liver transplantation, Yes vs No | 10/186 (5.4%) | 4/95 (4.2%) | 3/43 (7.0%) | 17/324 (5.2%) | 0.79 |
| Steroid responsive vs. non-responsive ^b^ | 103/186 (55.4%) | 48/95 (50.5%) | 19/43 (44.2%) | 170/324 (52.5%) | 0.38 |

*Note: Recent corticosteroid use is defined as having started steroids within 30 days prior to admission up to 30 days after admission.
^a^ P-values for continuous variables are to test for overall differences in means among steroid type. P-values for categorical variables are from Pearson χ² tests of association.*

*^b^ Steroid responsiveness defined by Lille score at day 4 or 7 lower than 0.45.*

**Supplementary Table 2.** Comparison of adjusted Kaplan-Meier survival probability estimate at 30-days after admission for each steroid type (prednisolone, prednisone, and methylprednisolone) among patients with severe alcohol-associated hepatitis.

| **Statistic** | **Estimate (95% CI)** | **P-value** |
| --- | --- | --- |
| ***Unadjusted K-M estimates [1]*** | | |
| 30-day survival estimate (%) [2] | | 0.04 |
| Prednisolone | 79.4 (69.7, 86.4) | – |
| Prednisone | 90.8 (85.5, 94.3) | – |
| Methylprednisolone | 82.7 (67.1, 91.4) | – |
| ***Adjusted K-M Estimates [2,3]*** | | |
| 30-day survival estimate (%) | | 0.12 |
| Prednisolone | 80.2 (71.5, 89.0) | – |
| Prednisone | 90.1 (85.7, 94.5) | – |
| Methylprednisolone | 84.4 (74.2, 94.7) | – |
| Absolute risk difference [p.p.] | | – |
| Prednisone vs Prednisolone | 9.9 (-0.2, 20.0) | 0.05 |
| Methylprednisolone vs Prednisolone | 4.2 (-9.7, 18.1) | 0.55 |
| Prednisone vs Methylprednisolone | 5.7 (-5.4, 16.8) | 0.31 |
| Relative risk of 30-day survival [2] | | 0.26 |
| Prednisone vs Prednisolone | 1.51 (0.85, 2.68) | 0.16 |
| Methylprednisolone vs Prednisolone | 1.10 (0.57, 2.10) | 0.77 |
| Prednisone vs Methylprednisolone | 1.37 (0.82, 2.30) | 0.23 |

*p.p. = percentage points
[1] Unadjusted survival estimates correspond to the K-M survival curve at 30-days.
[2] The p-value corresponds to an omnibus test for overall differences between steroid types.
[3] The model is a general linear model of pseudo-values with identity link (for absolute probability estimates and their differences) or a generalized linear model with the complementary log-log link for risk ratios. Both models use robust variance estimates, and are adjusted for age, sex, cirrhosis status at admission, MELD score at admission and acute kidney injury at admission.*
